# Quantitative Assessment of Gait Characteristics in Degenerative Cervical Myelopathy: A Prospective Clinical Study

**DOI:** 10.3390/jcm9030752

**Published:** 2020-03-10

**Authors:** Sukhvinder Kalsi-Ryan, Anna C. Rienmueller, Lauren Riehm, Colin Chan, Daniel Jin, Allan R. Martin, Jetan H. Badhiwala, Muhammad A. Akbar, Eric M. Massicotte, Michael G. Fehlings

**Affiliations:** 1 KITE-UHN, Toronto, ON M5G 2A2, Canada; Sukhvinder.Kalsi-Ryan@uhn.ca; 2 Department of Physical Therapy, University of Toronto, Toronto, ON M5G 1V7, Canada; 3 Spine Program; Krembil Brain Institute; University Health Network, Toronto, ON M5T 2S8, Canada; anna.rienmuller@mail.utoronto.ca (A.C.R.); lauren.riehm@medportal.ca (L.R.); cchan827@uwo.ca (C.C.); allan.martin@mail.utoronto.ca (A.R.M.); jetan.badhiwala@mail.utoronto.ca (J.H.B.); muhammad.akbar@mail.utoronto.ca (M.A.A.); Eric.Massicotte@uhn.ca (E.M.M.); 4 Department of Surgery and Spine Program, University of Toronto, Toronto, ON M5T 1P5, Canada; 5 Department of Orthopedic Surgery and Traumatology, Medical University Vienna, 1090 Vienna, Austria; 6 Department of Kinesiology, University of Waterloo, Waterloo, ON N2L 3G1, Canada; drqjin@edu.uwaterloo.ca

**Keywords:** degenerative cervical myelopathy, physical impairment, gait, locomotion, gait assessment, enhanced gait variability index

## Abstract

It is challenging to discriminate the early presentation of Degenerative Cervical Myelopathy (DCM) as well as sensitively and accurately distinguishing between mild, moderate, and severe levels of impairment. As gait dysfunction is one of the cardinal symptoms of DCM, we hypothesized that spatiotemporal gait parameters, including the enhanced gait variability index (eGVI), could be used to sensitively discriminate between different severities of DCM. A total of 153 patients recently diagnosed with DCM were recruited and stratified on the basis of DCM severity grades, as measured using the modified Japanese Orthopedic Association (mJOA) scale. Demographic information and neurological status were collected. Gait assessments were performed using an 8 m walkway. Spearman rank correlation was used to identify relationships between gait parameters and mJOA values as well as the mJOA lower extremity (LE) subscore. Kruskal–Wallis H test was performed to evaluate differences between severity groups, as defined by mJOA classification. A significant and relatively strong correlation was found between the mJOA score and eGVI, as well as between the LE subscore of the mJOA and eGVI. Significant differences in the eGVI (X^2^(2, N = 153) = 55.04, *p* < 0.0001, ε2 = 0.36) were found between all groups of DCM severity, with a significant increase in the eGVI as DCM progressed from mild to moderate. The eGVI was the most discriminative gait parameter, which facilitated objective differentiation between varying severities of DCM. Quantitative gait assessments show promise as an accurate and objective tool to diagnose and classify DCM, as well as to potentially evaluate the impact of therapeutic interventions.

## 1. Introduction

Degenerative Cervical Myelopathy (DCM) is a disorder involving chronic compression of the cervical spinal cord and is the most common form of spinal cord impairment in adults [1]. DCM can result from a wide range of pathologies, including degenerative disc disease, spondylosis, and hypertrophy or ossification of the spinal ligaments [2,3,4,5,6]. Cervical cord compression leads to nerve damage over time, resulting in loss of function and reduced quality of life [3,4,7,8]. Patients diagnosed with DCM usually present with at least one of the following symptoms: weakness and/or numbness of the upper extremities, reduced manual dexterity, gait and balance impairment, lower extremity spasticity, neuropathic pain, and bowel/bladder dysfunction. Although DCM is common, its detection can be challenging, as impairment can be quite subtle during the mild stage of the disease. 

Early diagnosis and management of DCM are important to accord appropriate care for those living with the condition. Current clinical methods for diagnosing DCM in the early stage or when the patient presents with mild symptoms are limited to subjective history taking and clinical assessment. Objective gait assessment can potentially detect early impairment. During gait, the center of mass is propelled forward as the body alternates between periods of single and double support, which produces challenges to the overall stability of the individual. While healthy adults can successfully walk with little difficulty, one of the cardinal symptoms of DCM is impaired gait [9,10,11]. In DCM, gait impairment is believed to be multifactorial, including upper motor neuron and proprioceptive dysfunction. The exact mechanisms have yet to be elucidated. However, the rubrospinal, reticulospinal, and vestibulospinal tracts are descending tracts that play a role in the stability of posture and gait and are likely implicated in DCM [12,13,14]. Gait impairment, particularly in the early stages of DCM, often presents as subtle instability in gait and balance, rather than gross and obvious impairments related to weakness or spasticity. 

Clinically, DCM is classified using the modified Japanese Orthopaedic Association scale (mJOA) [15]; with the lower extremity subscore of the mJOA describing gait impairment. The parameters that define these subtle deficits in gait are quite different from the spatiotemporal parameters that typically uncover gait impairment related to stroke or musculoskeletal issues. Therefore, we aimed to characterize the gait impairment of study participants with DCM to define and detect the specific changes resulting from progressive cervical spinal cord compression. There is evidence that individuals with moderate and severe DCM demonstrate slower gait speed, prolonged double support time, and reduced cadence, as compared to individuals lacking any physical impairments [10,16]. These adaptations serve to increase stability in DCM patients and to lower the risk of falling. Current literature has focused on either kinematics and gait parameters in patients with DCM requiring surgical intervention [10,17] or on postoperative walking speed [18]. It was shown that patients with DCM receiving conservative treatment have a significantly slower walking speed over time when compared to a surgical treatment group. Also, aberrant spinal alignment, including reduced cervical lordosis, head flexion, and increased anterior pelvic tilt documented in DCM patients preoperatively, lead to altered biomechanics of the lower extremities and therefore reduced walking speed, shorter stride length and stride time, as well as increased double support time [17]. Those studies involved patients with symptoms of myelopathy requiring surgical intervention. As delayed diagnosis and treatment might lead to greater disability [19], it seems to be important to focus on the early stages of myelopathy and on diagnostic tools. To date, there is no literature available which assesses gait parameters in patients with early or mild DCM and compares them to those of patients with more advanced DCM.

The objective of this study was to assess the correlation between subjective gait impairment of patients diagnosed with DCM, measured using the mJOA score and the lower extremity subscore, with objective gait parameters. Furthermore, we wanted to characterize mild, moderate, and severe DCM, as defined by the mJOA classification system, using quantitative spatiotemporal measurements of gait.

## 2. Materials and Methods 

### 2.1. Study Design

We conducted a single-center, observational, cross-sectional study involving 153 patients recently diagnosed with DCM between May 2013 and December 2017. Research ethics board approval was obtained, and all participants provided informed consent before participation. Inclusion criteria for this study were the following: (1) one or more clinical signs of DCM (corticospinal motor deficits, hand atrophy, hyperreflexia, a positive Hoffman sign, upgoing plantar reflexes, lower limb spasticity, and/or gait ataxia), (2) one or more clinical symptoms of DCM (numb hands, clumsy hands, gait impairment, bilateral hand paresthesia, L’Hermitte’s phenomenon, and/or weakness), and (3) MR imaging showing flattening, indentation, or circumferential compression of the spinal cord. Patients with previous cervical spine surgery, other documented neurological disease affecting gait assessment, disability of the lower extremities, or symptomatic lumbar stenosis and a Berg Balance Scale (BBS) <40 were excluded from the study. DCM severity was determined using the modified mJOA, and DCM was classified as mild, moderate or severe [1,2]. Demographic information, neurological examination, and BBS results to assess static and dynamic instability were documented. The control group comprised 13 healthy subjects without gait disorders, matched for age and gender, with a mean age of 56.8 ± 6.8 years. Gait data acquired from the healthy controls were used to calculate baseline values for spatiotemporal gait parameters. 

### 2.2. Scores

The mJOA consists of four categories with a maximum of 18 possible points: upper extremity motor dysfunction (5 possible points), lower extremity motor dysfunction (7 points, see Table 1), sensory impairment of the upper limbs (3 points), and bladder dysfunction (3 points). The study participants were evaluated at initial diagnosis with a score of 18, representing no functional deficit [15]. Mild DCM was defined by mJOA values between 15 and 17, moderate DCM by mJOA values from 12 to 14, and severe DCM by a mJOA score <12. [20].

The BBS measures balance impairment through 14 items scored from 0 to 4 points each and measures explicitly unsupported standing and sitting balance, as well as transfers [21]. A BBS of 40 has been used as a cut-off for independent ambulation [21].

### 2.3. Gait Assessment

**Figure 1:**
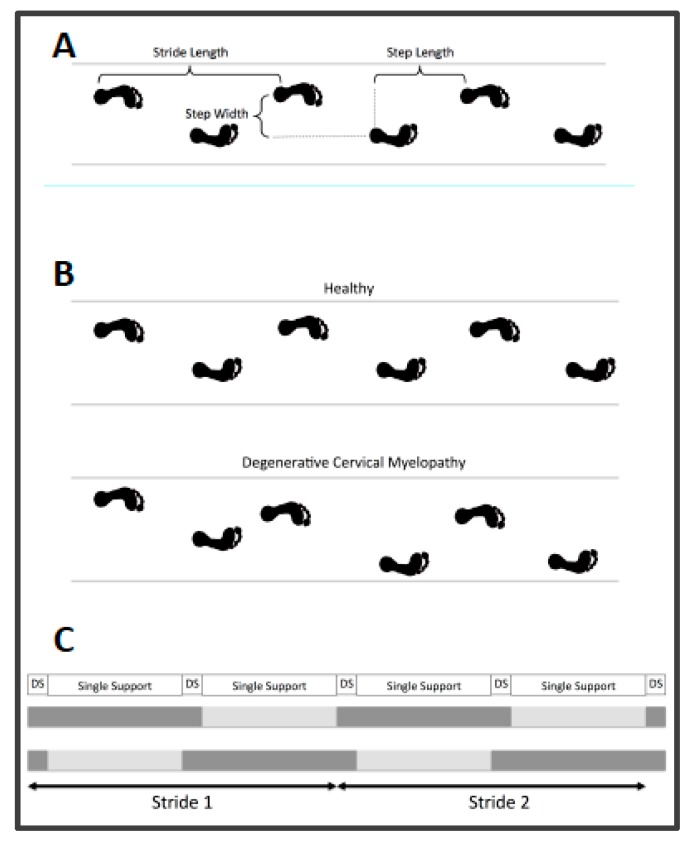
Visualization of spatial gait parameters (**A**). Visualization of gait variability in healthy subjects and increased variability in degenerative cervical myelopathy patients (**B**). Visualization of temporal gait parameters (**C**).

Gait assessment was performed in a standardized way for all participants. After careful instruction and a “warming-up” walk back and forth, patients were asked to walk across an 8-m walkway with an integrated pressure mat four times, barefoot and at a self-selected pace. Walking aids were not allowed. All gait assessments were conducted using either the GAITRite [22] (122 subjects, Franklin, NJ, USA) or the ProtoKinetics Zeno Walkway [23] (32 subjects, 13 control group subjects, Havertown, PA, USA). ProtoKinetics Movement Analysis (PKmas) software version 5.08C3i1 (Havertown, PA, USA) was used to collect gait data from both walkway systems; this software has been previously validated against the GAITRite walkway system [3]. Spatiotemporal gait parameters are presented in Table 2 and Figure 1.

### 2.4. Enhanced Gait Variability Index

The enhanced gait variability index (eGVI) is an improved version of the gait variability index, including a composite of measures of gait variability based on measured spatiotemporal parameters [24]. It is used to assess the quality of gait. Gait variability is defined as the fluctuation of gait measures between steps. This measure quantifies the amount of variability observed in an individual and compares it to that of a reference group. Five spatiotemporal parameters are taken into account for the calculation of eGVI: step length, step time, stance time, single-stance time, stride velocity. The weighted variability is then transformed into a score, with 100 representing the mean gait variability, and 10 representing 1 standard deviation from the mean in a reference population [25]. The gait variability index correlates well with clinical outcomes [26]. The eGVI is an advanced version of the GVI after correction of the directional specificity and magnitude problems detected when using the GVI in assessing GV [24]. The eGVI score was calculated as an average of the left and right variability index using the ProtoKinetics Movement Analysis (PKmas) software version 5.08C3i1 (Havertown, PA, USA). 

### 2.5. Statistics

Descriptive statistics were conducted for all parameters and are presented in mean ± SD.

**Figure 2:**
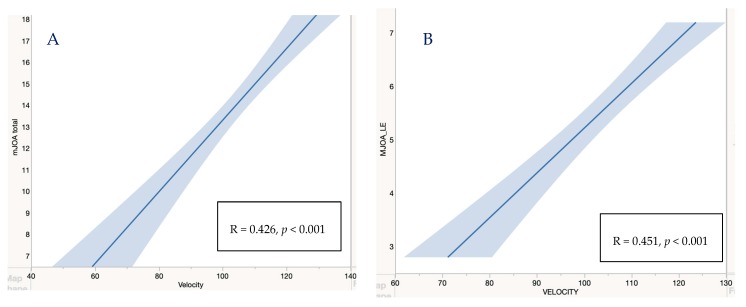
We observed a significant decrease in velocity with decreased mJOA score (**A**) and decreased mJOA LE subscore (**B**).

Shapiro Wilk test was used to test for normality. Levene’s test was used to assess the homogeneity of variance. To identify differences between DCM severity groups and acquired normative data, a one-way Kruskal–Wallis H test was conducted. A post-hoc test with Bonferroni correction was performed in the case of significance. Epsilon square was used as an effect size to indicate the magnitude of the difference between the severity groups. Spearman’s rank correlation coefficient was used to identify relationships between quantitative gait parameters and both the mJOA values as well as the mJOA lower extremity subscore (see Figure 2). The significance level was set at *p* ≤ 0.05. Statistical analysis was performed using R Version 3.6.1.

## 3. Results

### 3.1. Patient Demographics

The sample of DCM patients consisted of 83 male and 70 female participants, with a mean age of 56.81 ± 10.92 years. The mean duration of symptoms was 44.19 ± 56.06 months prior to assessment. Table 3 defines the sample stratified by mJOA into mild, moderate, and severe groups, also reporting the mean values and standard deviations of spatiotemporal gait parameters and eGVI. We found that 48.7% of patients in the mild DCM group, 21.2% in the moderate DCM group, and 0% in the severe DCM group presented within the range of eGVI of our control group. In addition, 35.9% of patients with mild DCM, 5.0% with moderate DCM, and 0% with severe DCM presented step length within the range of the control group.

### 3.2. Quantitative Assessment of Gait Parameters

Table 4 shows the correlation between gait parameters and mJOA values as well as mJOA lower extremity subscores. A significant relatively strong correlation was found between the subjective mJOA lower extremity subscore and eGVI (|R| = 0.567, *p* < 0.05) as well as velocity (|R| = 0.456, *p* < 0.05). Also, a significant relatively strong correlation was found between mJOA score and eGVI (|R| = 0.551, *p* < 0.05). A significant but moderate correlation was found between mJOA score and velocity (|R| = 0.426, *p* < 0.05), as shown in Table 4 and Figure 2.

**Figure 3:**
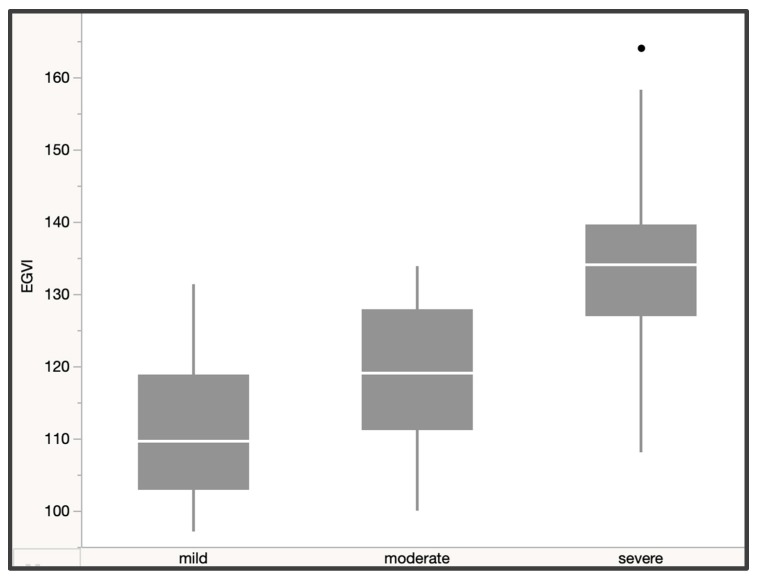
A significant increase in gait variability as measured by the eGVI was observed between severity groups in degenerative cervical myelopathy (DCM) patients.

The Kruskal–Wallis test showed a significant difference in gait variability (X^2^(2, N = 153) = 55.04, *p* < 0.0001, ε^2^ = 0.36). A post-hoc test using Dunn’s test with Bonferroni correction showed a significant increase in variability for more severe stages of DCM (*p* < 0.001) and a strong effect size (ε^2^ = 0.36). We found a mean score of 111.18 ± 9.85 for mild DCM versus a mean score of 119.14 ± 10.14 for moderate DCM (mild/moderate = *p* < 0.001) and a mean of 132.94 ± 12.78 for severe DCM (moderate/severe = *p* < 0.001). We also detected a significant difference in velocity (X^2^(2, N = 153) = 35.59, *p* < 0.0001, ε^2^ = 0.23), stride velocity (X^2^(2, N = 153) =32.79, *p* < 0.0001, ε^2^ = 0.22), and step length (X^2^(2, N = 153) = 30.23, *p* < 0.0001, ε^2^ = 0.19) between patients with moderate and severe DCM, as shown in Table 5 and Figure 3.

## 4. Discussion

To our knowledge, this is the largest study to date characterizing specific differences in gait parameters between severity subtypes of DCM. Additionally, this study assessed the correlation between objective spatiotemporal gait parameters and subjective clinical gait impairment in patients with DCM.

We found significant differences between control subjects and patients with mild, moderate, and severe DCM. Specifically, the enhanced gait variability index proved to be a useful tool to document significant differences between all severity groups as defined by the mJOA. Mean eGVI increased significantly from 103.36 ± 4.54 in the control group to 110.9 ± 9.73 in patients with mild DCM, 119.14 ± 10.14 in patients with moderate DCM, and 132.94 ± 12.78 in patients with severe DCM. Based on the literature [24], an eGVI of approximately 100 is within normative range, and a clinically relevant difference occurs when there is a change of at least 10 points (one SD). 

Gait deficits are commonly self-reported and, at times, objectively measured with timed walking tests [18]. The primary screening tool used to evaluate individuals with DCM is the mJOA scale [15], which is a subjective clinical score that also assesses walking difficulties. The mJOA score is used to stratify DCM by severity, with mild DCM represented by a score of 15–17, moderate DCM by a score of 12–14, and severe DCM by a score <12 [20]. The lower extremity subscore is presented in Table 3 and Figure 2. Patients with mild DCM commonly report only minimal gait impairment, and in these cases, gait deficits are typically not detectable with routine clinical exams [20,27]. Timed walking tests can detect changes in gait speed; however, for individuals with mild DCM, gait velocity typically falls within a normative range, meaning that subtle impairments cannot be quantified. While the subtle deficits do not have a definitive impact on function, identifying these changes can be essential for early identification of the disease and monitoring disease progression. 

In contrast to the large amount of literature surrounding gait analysis in other neurological conditions [28], such as stroke [29], Parkinson’s disease, and other neurological conditions [26], little is known about specific spatiotemporal gait parameters in DCM [9,28]. In a recent publication, Zheng et al. [30] evaluated the correlation between the JOA score and specific gait parameters in patients with DCM and lumbar disc herniation (LDH). They found only a weak correlation between the JOA score and step duration, cycle duration, double-support time, gait speed, cadence, and stride length and no correlation with single-support time. In a multiple regression analysis, they only found the lower extremity motor function subscore as a significant but weakly correlated parameter, but no significant factor was associated with the motor function of lower extremities. In contrast, our study shows a significant and moderately strong correlation between the mJOA score and both velocity and step length, as well as a relatively strong correlation between the mJOA score and the eGVI. Zheng et al. [30] state that the JOA scoring system might not adequately reflect gait impairment and that gait analysis might be more reliable in detecting walking impairment. Since they found a better correlation between the JOA lumbar score and gait parameters, they suggested that the difference might be due to the use of fewer questions regarding walking in the JOA cervical score. In contrast, we were able to demonstrate a significant correlation between the mJOA score and various spatiotemporal gait parameters. This might be due to the use of the mJOA scale in our study, where the emphasis on walking was improved. 

We also found significantly reduced velocity, stride velocity, and step length between moderate and severe DCM groups. Singh et al. found a continuous decrease in walking speed with time after the initial diagnosis of DCM and a significantly increased walking speed after cervical decompressive surgery [18] using a 30-m walking test. In comparison, Haddas et al. [17] found significantly decreased cadence, velocity, single-support time, step length, and step width in patients with DCM compared to a healthy control group. They only assessed patients already scheduled for decompression surgery, with more severe DCM, which explains the higher correlation in most gait parameters. Decreased velocity, step velocity, and step length, are most likely related to decreased balance while walking as DCM progresses. Reduced velocity and step length will help increase gait stability and, therefore, also decrease gait variability. This might cover part of the variability in gait parameters, especially at mild stages of DCM, and explains the different results in comparison to those of Haddas et al. 

This study has several limitations. Patients in the severe DCM group were significantly older than patients in the other two groups (mean age of 62 in severe DCM versus a mean age of 55 in mild and moderate DCM). This age difference might contribute to further changes in gait assessment in comparison to younger subjects. Virmani et al. [31] were able to show a significantly increased variability in stride length with age during steady-state gait using stepwise multiple regression analysis. When using univariate analysis, an increase in stride velocity was also detected. Velocity and stride velocity also significantly decreased with more severe DCM in our dataset. This was observed not only when comparing patients with moderate and severe DCM, but also when comparing the control group with patients with mild and moderate DCM, although the last three groups did not present a significant age difference. Another drawback is that we used average data between the left and the right leg, which might hide relevant information, especially in relation to variability assessments, but at the advantage of eliminating lower extremity-related gait patterns. 

Further research is necessary to evaluate and compare pre- and postoperative/post-treatment gait parameters in patients diagnosed with DCM. This can provide further insight into subtle changes associated with disease progression and treatments. The authors believe this work is only the initial step in defining a sensitive assessment that can characterize gait impairment in DCM patients. We look to continuing developing these findings into more validated and psychometrically sound parameters as the measures continue to be used and implemented in clinical/research environments.

## Figures and Tables

**Table 1:** Lower extremity subscore of modified Japanese Orthopaedic Association scale (mJOA).

Lower Extremity Subscore (/7)	0	Complete loss of movement and sensation
	1	Complete loss of movement, some sensation present
	2	Inability to walk, but some movement
	3	Able to walk on flat ground with walking aid
	4	Able to walk without walking aid but must hold a handrail on stairs
	5	Moderate to severe walking imbalance, but able to perform stairs without handrail
	6	Mild imbalance when standing OR walking
	7	Normal walking

**Table 2:** Spatiotemporal gait parameters.

Parameter	Description	Unit
Velocity	Walking speed = distance per time	cm/s
Cadence	Steps per minute	steps/min
Base of support	Step width = perpendicular distance between two points on both feet measured during two consecutive steps	m
Step length	Distance between ground contact of one foot and the next subsequent ground contact of the opposite foot in the direction of progression	m
Stride length	Distance between ground contact of one foot and the next subsequent ground contact of the same foot in the direction of progression	m
Step time	Time between ground contact of one foot and the next subsequent ground contact of the opposite foot	s
Single-stance time	Time during gait cycle while one foot is on the ground	s
Double-stance time	Time during gait cycle while two feet are on the ground	s
Total stance time	Time that passes during single and double support of the stance phase of one extremity during a gait cycle	s
eGVI	enhanced gait variability index (includes 5 spatiotemporal gait parameters: step time, step length, step velocity, total stance time, single-stance time)	

**Table 3:** Mean (± SD) of patient and gait specific parameters, stratified by the modified Japanese Orthopaedic Association (mJOA) scale. mJOA LE: mJOA lower extremity.

Variable	Control Group n = 13	Mild DCM n = 82	Moderate DCM n = 40	Severe DCM n = 31	All DCM n = 153
Age	56.75 (6.77)	55.3 (11.01)	55.73 (9.75)	62.19 (10.91)	56.81 (10.92)
mJOA Score		15.92 (0.73)	13.13 (0.82)	9.94(2.5)	13.98 (2.50)
mJOA LE Subscore		6.51 (0.55)	5.10 (1.12)	3.71 (1.35)	5.58 (1.35)
Berg Balance Score		53.52 (5.24)	49.63 (7.09)	42.59 (4.65)	47(6.1)
Velocity (cm/sec)	119.22 (11.61)	114.84 (23.71)	106.44 (23.72)	74.18 (29.51)	104.41 (29.51)
Cadence (steps/min)	114.74(9.49)	111.49 (12.67)	108.58 (12.46)	92.55(15.98)	106.89 (15.99)
Base of Support (cm)	8.16 (3.74)	9.13 (3.28)	8.21 (3.85)	9.24 (3.65)	8.91 (3.65)
Step Length (cm)	63.57 (4.87)	60.81 (9.66)	57.94 (8.83)	45.96 (11.44)	57.05 (11.44)
Total Stance Time (sec)	0.649 (0.12)	0.702 (0.10)	0.717 (0.09)	0.905 (0.25)	0.747 (0.163)
Single-Support Time (sec)	0.410 (0.03)	0.389 (0.04)	0.395 (0.04)	0.409 (0.07)	0.394 (0.046)
Double-Support Time (sec)	0.249 (0.029)	0.303 (0.08)	0.316 (0.06)	0.485 (0.22)	0.343 (0.138)
Single-Stance Ratio	1.56 (0.20)	1.35 (0.29)	1.29 (0.23)	0.99 (0.38)	1.26 (0.32)
Enhanced Gait Variability Index	103.36(4.54)	110.9 (9.73)	119.14 (10.14)	132.94 (12.78)	117.54 (13.5)

**Table 4:** Spearman’s rank correlation coefficients between gait parameters compared with mJOA LE subscore and mJOA score (total). Confidence interval was set to 95%.

Gait Parameters	mJOA LE	*p*−Value	mJOA	*p*−Value
Velocity (cm/sec)	0.456	<0.001	0.426	<0.001
Cadence (steps/min)	0.346	<0.001	0.286	<0.001
Base of Support (cm)	0.044	0.6	0.038	0.6
Step Length (cm)	0.434	<0.001	0.417	<0.001
Total Stance Time (sec)	−0.352	<0.001	−0.303	<0.001
Single-Support Time (sec)	−0.058	0.47	0.004	0.959
Double-Support Time (sec)	−0.404	<0.001	−0.382	<0.001
Single-Stance Ratio	0.413	<0.001	0.417	<0.001
Enhanced Gait Variability Index	−0.567	<0.001	−0.551	<0.001

**Table 5:** Kruskal–Wallis H-test, Bonferroni-adjusted *p*, and Epsilon squared effect sizes.

Gait Parameter	H(df)	*p*	Padj Mild/Moderate	Padj Mild/Severe	Padj Moderate/Severe	Epsilon^2^
Velocity	35.59(2)	<0.0001	0.081	<0.0001	0.001	0.23 ^+^
Cadence	22.92(2)	<0.0001	0.59	<0.0001	0.004	0.15
Base of support	2.73(2)	0.26	-	-	-	0.02
Step Length	30.23(2)	<0.0001	0.25	<0.0001	0.002	0.19 ^+^
Stride Velocity	32.79(2)	<0.0001	0.08	<0.0001	0.003	0.22 ^+^
Total Stance Time	21.80(2)	0.0002	0.72	<0.0001	0.005	0.14
Single-Support Time	1.83(2)	0.4	-	-	-	0.01
Double-Support Time	25.54(2)	<0.0001	0.34	<0.0001	0.0043	0.16
Single-Stance Ratio	25.96(2)	<0.0001	0.59	<0.0001	0.002	0.17
eGVI	55.04(2)	<0.0001	0.001*	<0.0001 *	0.001 *	0.36 ^++^
Age	9.22(2)	0.01	1	0.012	0.023	0.06

* significant difference, ^++^ strong effect size, ^+^ relatively strong effect size.
